# Associations Among Dementia, Physical Activity, and Sedentary Behavior: Different Roles of Total and Sub-domains

**DOI:** 10.2188/jea.JE20220148

**Published:** 2023-08-05

**Authors:** Yoshio Nakata

**Affiliations:** Faculty of Health and Sport Sciences, University of Tsukuba, Ibaraki, Japan

**Keywords:** exercise, leisure activities, cognitive dysfunction, surveys and questionnaires

Physical activity (PA) is well known to lower the risk of dementia. A recent systematic review and meta-analysis including 58 prospective cohort and case-control studies showed that baseline PA was associated with a decreased risk of all-cause dementia (pooled relative risk 0.80; 95% confidence interval [CI], 0.77–0.84).^1^ Sedentary behavior (SB) is a relatively newly-identified risk factor of dementia. A systematic review and meta-analysis including 18 cohort studies revealed that SB was associated with an increased risk of dementia (relative risk 1.30; 95% CI, 1.12–1.51).^2^ Therefore, promoting PA and reducing SB could be possible preventative measures for dementia.

A recent study from the Japan Public Health Center-based Prospective Disabling Dementia Study reported an association between PA and the risk of developing disabling dementia.^3^ This study followed 43,896 participants (mean age 61.0; standard deviation [SD], 7.5 years at baseline survey) for a mean of 9.5 (SD, 2.8) years and found that the highest total PA group exhibited a lowered risk of dementia compared with the lowest PA group (adjusted hazard ratio 0.75; 95% CI, 0.66–0.85 in men and 0.75; 95% CI, 0.67–0.84 in women). Similarly, moderate-to-vigorous PA (MVPA) and leisure-time MVPA were negatively associated with the risk of dementia. However, after controlling for potential reverse causation bias with the sequential exclusion of incidents of disabling dementia arising each year from the first year of the dementia ascertainment period, the significant associations of total PA and MVPA with dementia disappeared. Significant associations between leisure-time MVPA remained in men but not in women. The authors discussed the possible role of leisure activities, including cognitive training, which have a protective function against dementia. Therefore, an assessment of total PA alone might not be sufficient to illustrate the association between PA and dementia.

In this issue, Nemoto et al^4^ reported the association of SB with dementia. Interestingly, they compared the associations between mentally active SB (MASB) and passive SB (PSB). MASB was operationally defined as reading books or newspapers, while PSB was defined as TV viewing. MASB was classified as low (<10 min/day), moderate (10–30 min/day), or high (>30 min/day), and PSB was classified as low (<1 h/day), moderate (1–3 h/day), or high (>3 h/day). In addition, they evaluated PA using the International Physical Activity Questionnaire (IPAQ)^5^ and categorized PA as either low (<2.5 metabolic equivalent of task [MET]-h/week), moderate (2.5–16.0 MET-h/week), or high (≥16.0 MET-h/week). In this study, 5,323 older adults were followed for 5 years, and the results showed that the cumulative incidence of dementia was the highest in the low-PA, low-MASB, and high-PSB categories. However, the independent impact on dementia onset after adjusting for covariates was significant only for moderate or high PA levels and high MASB levels. PSB showed no impact on dementia onset across all PA levels. In addition, analysis of the joint impact of PA and MASB showed that MASB levels were not associated with dementia onset when PA levels were low. In contrast, moderate and high MASB levels were significantly associated with decreased dementia onset when PA levels were moderate or high. Therefore, the authors concluded that interventions to promote MASB and PA can be beneficial in delaying or preventing the onset of dementia.

Previous studies have shown that the increased time spent in SB could be attributed to the less time spent performing light-intensity PA (LIPA),^6^ and an almost perfect inverse correlation between LIPA and SB has been reported (Spearman’s rho = −0.98).^7^ Therefore, it seems easier to substitute SB with LIPA; however, it is more difficult to substitute SB with MVPA. To delay or prevent dementia onset, Nemoto et al^4^ recommended combining high PA levels and moderate or high MASB levels, which lowers the dementia risk by approximately 60% compared to low PA and low MASB levels. This recommendation corresponds to 30–40 min/day MVPA and >10 min/day MASB. These activities therefore occupy a relatively small part of waking hours, while the contribution of intentional behavior is shown to be large.

Nemoto et al^4^ raised some limitations of this study. First, PA and SB were self-reported. However, objectively measured PA and SB cannot be classified as leisure-time PA or MASB. Therefore, I believe that subjective questionnaires have different roles in PA and SB assessment. If the authors used the IPAQ-long^5^ or Global Physical Activity Questionnaire (GPAQ),^8^ the contribution of leisure-time MVPA or other domains of PA could be evaluated, as Ihira et al reported.^3^ An additional limitation was the shorter follow-up period. Ihira et al^3^ performed follow up for approximately 10 years to exclude the possibility of reverse causation. An adequate follow-up period may show a negative association between PSB and dementia onset. Residual confounding variables were another limitation of this study. Physical fitness was considered a potential residual covariate. Tin et al^9^ revealed the association of dual decline in memory and gait speed with the risk of dementia. Hatabe et al^10^ showed a negative association of handgrip strength with dementia in the Hisayama study. Gait velocity decline was also reported to be associated with the risk of incident dementia.^11^ The authors raised genetic factors and other health behaviors as residual covariates. Further studies are needed to address these limitations.
